# Dicyanidobis(*N*,*N*′-dimethythio­urea-κ*S*)mercury(II)

**DOI:** 10.1107/S1600536810030424

**Published:** 2010-08-04

**Authors:** Muhammad Riaz Malik, Saqib Ali, Saeed Ahmad, Muhammad Altaf, Helen Stoeckli-Evans

**Affiliations:** a Department of Chemistry, Quaid-i-Azam University, Islamabad, Pakistan; b Department of Chemistry, University of Engineering and Technology, Lahore 54890, Pakistan; c Institute of Physics, University of Neuchâtel, rue Emile-Argand 11, CH-2009 Neuchâtel, Switzerland

## Abstract

In the title complex, [Hg(CN)_2_(C_3_H_8_N_2_S)_2_], the Hg^II^ atom is located on a twofold rotation axis. It is four-coordinate having an irregular tetra­hedral geometry composed of two cyanide C atoms [Hg—C = 2.090 (6) Å] and two thione S atoms of *N*,*N*′-dimethyl­thio­urea (dmtu) [Hg—S = 2.7114 (9) Å]. The NC—Hg—CN bond angle of 148.83 (13)° has the greatest deviation from the ideal tetra­hedral geometry. The mol­ecular structure is stabilized by intra­molecular N—H⋯S inter­actions involving dmtu units related by the twofold symmetry. In the crystal, inter­molecular N—H⋯N(CN) hydrogen-bonding inter­actions link symmetry-related mol­ecules into a two-dimensional network in (110).

## Related literature

For the biological applications of mercury(II) complexes of thi­o­nes, see: Akrivos (2001 ▶); Bell *et al.* (2001 ▶); Popovic *et al.* (2000 ▶). For background to mercury(II) complexes of thio­urea and its derivatives, see: Ahmad *et al.* (2009 ▶); Jiang *et al.* (2001 ▶); Lobana *et al.* (2008 ▶); Mufakkar *et al.* (2010 ▶); Nawaz *et al.* (2010 ▶); Popovic *et al.* (2000 ▶); Wu *et al.* (2004 ▶). For the crystal structures of cyanide complexes of *d* ^10^ metals, see: Ahmad *et al.* (2009 ▶); Altaf *et al.* (2010 ▶); Fettouhi *et al.* (2010 ▶); Hanif *et al.* (2007 ▶).
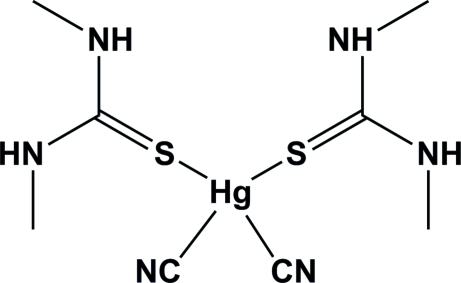


## Experimental

### 

#### Crystal data

[Hg(CN)_2_(C_3_H_8_N_2_S)_2_]
*M* *_r_* = 460.98
Monoclinic, 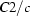

*a* = 18.1161 (11) Å
*b* = 7.7533 (5) Å
*c* = 14.0553 (8) Å
β = 128.533 (3)°
*V* = 1544.32 (16) Å^3^
*Z* = 4
Mo *K*α radiation
μ = 10.23 mm^−1^
*T* = 173 K
0.40 × 0.31 × 0.25 mm

#### Data collection

Stoe IPDS 2 diffractometer
Absorption correction: multi-scan (*MULscanABS* embedded in *PLATON*; Spek, 2009 ▶) *T*_min_ = 0.270, *T*_max_ = 1.000
8116 measured reflections
1451 independent reflections
1411 reflections with *I* > 2σ(*I*)
*R*_int_ = 0.049

#### Refinement

*R*[*F* ^2^ > 2σ(*F* ^2^)] = 0.017
*wR*(*F* ^2^) = 0.036
*S* = 1.14
1451 reflections
88 parameters
H atoms treated by a mixture of independent and constrained refinement
Δρ_max_ = 0.68 e Å^−3^
Δρ_min_ = −1.97 e Å^−3^

### 

Data collection: *X-AREA* (Stoe & Cie, 2009 ▶); cell refinement: *X-AREA*; data reduction: *X-RED32* (Stoe & Cie, 2009 ▶); program(s) used to solve structure: *SHELXS97* (Sheldrick, 2008 ▶); program(s) used to refine structure: *SHELXL97* (Sheldrick, 2008 ▶); molecular graphics: *PLATON* (Spek, 2009 ▶); software used to prepare material for publication: *SHELXL97* and *PLATON*.

## Supplementary Material

Crystal structure: contains datablocks I, global. DOI: 10.1107/S1600536810030424/wm2389sup1.cif

Structure factors: contains datablocks I. DOI: 10.1107/S1600536810030424/wm2389Isup2.hkl

Additional supplementary materials:  crystallographic information; 3D view; checkCIF report

## Figures and Tables

**Table 1:** Hydrogen-bond geometry (Å, °)

*D*—H⋯*A*	*D*—H	H⋯*A*	*D*⋯*A*	*D*—H⋯*A*
N1—H1*N*⋯S1^i^	0.80 (6)	2.67 (5)	3.415 (4)	157 (4)
N2—H2*N*⋯N3^ii^	0.79 (5)	2.21 (6)	2.951 (7)	155 (4)

Symmetry codes: (i) 
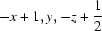
; (ii) 
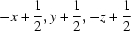
.

**Fig. 1.**
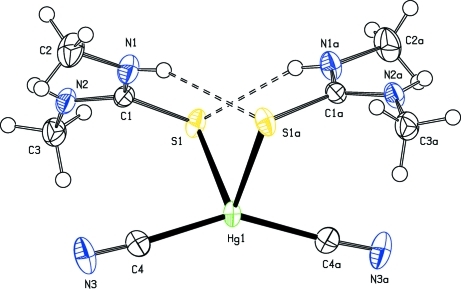
The molecular structure of the title compound with the atomic numbering scheme. Displacement ellipsoids are drawn at the 50% probability level. The intramolecular N—H···S interactions are shown as double dashed lines (see Table 1 for details). [Symmetry code (a) -x+1, y, -z+1/2.]

**Fig. 2.**
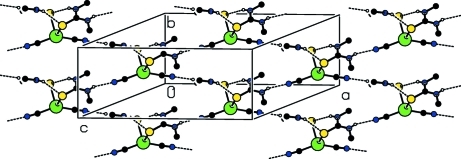
A crystal packing diagram of the title complex showing the N—H···S and N—H···N hydrogen bonding interactions (dashed lines; see Table 1 for details).

## supplementary crystallographic information

### Comment

The structural characterization of mercury(II) complexes of thioamides is an important aspect of inorganic chemistry because such complexes can be used as models for metal-sulfur interactions in biological systems (Akrivos, 2001; Bell *et al.*, 2001; Popovic *et al.*, 2000). Several crystallographic reports about mercury(II) complexes of the type, *L*_2_HgX_2_ (*L* = thiourea or its derivatives) reveal that these complexes usually consist of discrete monomeric molecules with tetrahedral (somewhat distorted) coordination environments around mercury(II) (Ahmad *et al.*, 2009; Bell *et al.*, 2001; Jiang *et al.*, 2001; Lobana *et al.*, 2008; Mufakkar *et al.*, 2010; Nawaz *et al.*, 2010; Popovic *et al.*, 2000; Wu *et al.*, 2004). Recently, we have reported the crystal structures of a number of cyanido complexes of *d*^10^ metal ions with *L*-type ligands, including the crystal structure of a trinuclear complex, [{(tmtu)_2_Hg(CN)_2_}_2_^.^Hg(CN)_2_] (tmtu = tetramethylthiourea), which presents a unique example of a Hg(CN)_2_ bridged mercury(II)-thione complex (Ahmad *et al.*, 2009; Altaf *et al.*, 2010; Fettouhi *et al.* 2010; Hanif *et al.*, 2007). Herein, we report on the crystal structure of the title mercury cyanide complex of *N,N*'-dimethylthiourea, [Hg(dmtu)_2_(CN)_2_].

The title monomeric complex is composed of an Hg(CN)_2_ unit with two *N,N*'-dimethylthiourea (dmtu) ligands coordinated to the Hg atom *via* the S atom (Fig. 1). The four-coordinate mercury atom is located on a two-fold rotation axis and adopts a severely distorted tetrahedral geometry, the bond angles being in the range of 94.31 (3) - 148.83 (13)°. The molecular structure is stabilized by intramolecular N-H···S interactions involving dmtu units related by the two-fold symmetry (Fig. 1, Table 1). The bond distances and bond angles are in agreement with those reported for related compounds (Ahmad *et al.*, 2009; Altaf *et al.*, 2010; Jiang *et al.*, 2001; Lobana *et al.*, 2008; Mufakkar *et al.*, 2010; Nawaz *et al.*, 2010; Popovic *et al.*, 2000; Wu *et al.*, 2004). The SCN_2_ moiety of dmtu is planar [to within 0.002 (1) Å] with the C—N and C—S bond lengths corresponding to the values intermediate between single and double bonds. The Hg-C≡N unit is nearly linear with a bond angle of 175.3 (3)°. The compound is closely related with [Hg(*N,N*'-dibutylthiourea)_2_(CN)_2_] (Ahmad *et al.*, 2009).

In the crystal packing of the title complex, symmetry-related molecules are connected *via* intermolecular N—H···N hydrogen bonds, involving the thiourea NH atoms and the N atom of the CN^-^ anions (Fig. 2, Table 1). This gives rise to the formation of a two-dimensional network in (110). This is the same arrangement as observed previously for the dibutylthiourea compound mentioned above.

### Experimental

To 0.25 g (1.0 mmol) mercury(II) cyanide in 10 ml methanol was added 2 equivalents of *N*,*N*'-dimethylthiourea in methanol. On mixing, a clear solution was obtained. It was then stirred for 30 minutes after which it was filtered and the filtrate kept at RT for crystallization by slow evaporation of the solvent. As a result, colourless block-like crystals, suitable for X-ray diffraction analysis, were obtained.

### Refinement

The NH H-atoms were located in difference electron-density maps and were freely refined: N—H = 0.80 (6) & 0.79 (5) Å. The C-bound H-atoms were included in calculated positions and treated as riding atoms: C—H = 0.98 Å, with *U*_iso_(H) = 1.5*U*_eq_(parent C-atom).

### Crystal data

**Table:**

[Hg(CN)_2_(C_3_H_8_N_2_S)_2_]	*F*(000) = 872
*M**_r_* = 460.98	*D*_x_ = 1.983 Mg m^−^^3^
Monoclinic, *C*2/*c*	Mo *K*α radiation, λ = 0.71073 Å
Hall symbol: -C 2yc	Cell parameters from 12331 reflections
*a* = 18.1161 (11) Å	θ = 1.9–26.1°
*b* = 7.7533 (5) Å	µ = 10.23 mm^−^^1^
*c* = 14.0553 (8) Å	*T* = 173 K
β = 128.533 (3)°	Block, colourless
*V* = 1544.32 (16) Å^3^	0.40 × 0.31 × 0.25 mm
*Z* = 4	

### Data collection

**Table:**

Stoe IPDS 2 diffractometer	1451 independent reflections
Radiation source: fine-focus sealed tube	1411 reflections with *I* > 2σ(*I*)
graphite	*R*_int_ = 0.049
φ– + ω– scans	θ_max_ = 25.6°, θ_min_ = 2.9°
Absorption correction: multi-scan (MULscanABS embedded in *PLATON*; Spek, 2009)	*h* = −21→21
*T*_min_ = 0.270, *T*_max_ = 1.000	*k* = −9→9
8116 measured reflections	*l* = −17→17

### Refinement

**Table:**

Refinement on *F*^2^	Primary atom site location: structure-invariant direct methods
Least-squares matrix: full	Secondary atom site location: difference Fourier map
*R*[*F*^2^ > 2σ(*F*^2^)] = 0.017	Hydrogen site location: inferred from neighbouring sites
*wR*(*F*^2^) = 0.036	H atoms treated by a mixture of independent and constrained refinement
*S* = 1.14	*w* = 1/[σ^2^(*F*_o_^2^) + (0.0129*P*)^2^ + 2.6833*P*] where *P* = (*F*_o_^2^ + 2*F*_c_^2^)/3
1451 reflections	(Δ/σ)_max_ < 0.001
88 parameters	Δρ_max_ = 0.68 e Å^−^^3^
0 restraints	Δρ_min_ = −1.97 e Å^−^^3^

### Special details

**Table:**

Geometry. Bond distances, angles *etc*. have been calculated using the rounded fractional coordinates. All su's are estimated from the variances of the (full) variance-covariance matrix. The cell e.s.d.'s are taken into account in the estimation of distances, angles and torsion angles
Refinement. Refinement of *F*^2^ against ALL reflections. The weighted *R*-factor *wR* and goodness of fit *S* are based on *F*^2^, conventional *R*-factors *R* are based on *F*, with *F* set to zero for negative *F*^2^. The threshold expression of *F*^2^ > σ(*F*^2^) is used only for calculating *R*-factors(gt) *etc*. and is not relevant to the choice of reflections for refinement. *R*-factors based on *F*^2^ are statistically about twice as large as those based on *F*, and *R*- factors based on ALL data will be even larger.

### Fractional atomic coordinates and isotropic or equivalent isotropic displacement parameters (Å^2^)

**Table:**

	*x*	*y*	*z*	*U*_iso_*/*U*_eq_	
Hg1	0.50000	−0.21587 (2)	0.25000	0.0231 (1)	
S1	0.37952 (6)	0.02195 (11)	0.08082 (7)	0.0255 (3)	
N1	0.3941 (2)	0.1650 (4)	0.2640 (3)	0.0277 (9)	
N2	0.2450 (2)	0.0856 (4)	0.1004 (2)	0.0241 (8)	
N3	0.3776 (3)	−0.3173 (4)	0.3370 (3)	0.0441 (12)	
C1	0.3355 (2)	0.0948 (4)	0.1535 (3)	0.0210 (9)	
C2	0.3657 (3)	0.2174 (5)	0.3367 (3)	0.0386 (13)	
C3	0.1748 (2)	0.0052 (5)	−0.0168 (3)	0.0306 (11)	
C4	0.4229 (3)	−0.2883 (4)	0.3084 (3)	0.0295 (10)	
H1N	0.449 (3)	0.161 (5)	0.296 (3)	0.024 (10)*	
H2A	0.31970	0.31150	0.29540	0.0580*	
H2B	0.42120	0.25690	0.41680	0.0580*	
H2C	0.33710	0.11910	0.34680	0.0580*	
H2N	0.227 (3)	0.128 (5)	0.134 (3)	0.030 (10)*	
H3A	0.17870	0.05510	−0.07770	0.0460*	
H3B	0.11180	0.02530	−0.04110	0.0460*	
H3C	0.18680	−0.11920	−0.01060	0.0460*	

### Atomic displacement parameters (Å^2^)

**Table:**

	*U*^11^	*U*^22^	*U*^33^	*U*^12^	*U*^13^	*U*^23^
Hg1	0.0209 (1)	0.0274 (1)	0.0294 (1)	0.0000	0.0197 (1)	0.0000
S1	0.0249 (5)	0.0344 (4)	0.0242 (4)	0.0067 (3)	0.0188 (4)	0.0020 (3)
N1	0.0244 (18)	0.0359 (17)	0.0275 (13)	0.0008 (14)	0.0185 (14)	−0.0045 (12)
N2	0.0225 (16)	0.0279 (15)	0.0273 (13)	0.0015 (11)	0.0181 (12)	−0.0035 (11)
N3	0.040 (2)	0.058 (2)	0.054 (2)	−0.0070 (17)	0.0389 (19)	0.0028 (16)
C1	0.0236 (18)	0.0192 (15)	0.0255 (14)	0.0042 (12)	0.0179 (14)	0.0031 (11)
C2	0.041 (3)	0.050 (2)	0.0352 (18)	−0.0019 (18)	0.0288 (19)	−0.0128 (16)
C3	0.022 (2)	0.0341 (19)	0.0336 (16)	−0.0025 (15)	0.0163 (16)	−0.0047 (14)
C4	0.028 (2)	0.0288 (17)	0.0344 (16)	−0.0018 (15)	0.0207 (16)	0.0009 (14)

### Geometric parameters (Å, °)

**Table:**

Hg1—S1	2.7114 (9)	N3—C4	1.139 (8)
Hg1—C4	2.090 (6)	N1—H1N	0.80 (6)
Hg1—S1^i^	2.7114 (9)	N2—H2N	0.79 (5)
Hg1—C4^i^	2.090 (6)	C2—H2A	0.9800
S1—C1	1.736 (4)	C2—H2B	0.9800
N1—C1	1.335 (5)	C2—H2C	0.9800
N1—C2	1.459 (7)	C3—H3A	0.9800
N2—C1	1.314 (6)	C3—H3B	0.9800
N2—C3	1.452 (4)	C3—H3C	0.9800
			
S1—Hg1—C4	99.05 (11)	S1—C1—N1	119.6 (3)
S1—Hg1—S1^i^	94.31 (3)	Hg1—C4—N3	175.3 (3)
S1—Hg1—C4^i^	102.01 (9)	N1—C2—H2A	109.00
S1^i^—Hg1—C4	102.01 (9)	N1—C2—H2B	110.00
C4—Hg1—C4^i^	148.83 (13)	N1—C2—H2C	109.00
S1^i^—Hg1—C4^i^	99.05 (11)	H2A—C2—H2B	109.00
Hg1—S1—C1	96.84 (11)	H2A—C2—H2C	109.00
C1—N1—C2	123.8 (4)	H2B—C2—H2C	109.00
C1—N2—C3	124.7 (3)	N2—C3—H3A	109.00
C1—N1—H1N	117 (3)	N2—C3—H3B	110.00
C2—N1—H1N	118 (3)	N2—C3—H3C	109.00
C3—N2—H2N	117 (3)	H3A—C3—H3B	110.00
C1—N2—H2N	118 (3)	H3A—C3—H3C	109.00
S1—C1—N2	121.1 (3)	H3B—C3—H3C	109.00
N1—C1—N2	119.3 (4)		
			
C4—Hg1—S1—C1	32.52 (15)	C2—N1—C1—S1	−174.9 (3)
S1^i^—Hg1—S1—C1	−70.39 (13)	C2—N1—C1—N2	6.6 (5)
C4^i^—Hg1—S1—C1	−170.60 (16)	C3—N2—C1—S1	4.6 (5)
Hg1—S1—C1—N1	60.6 (3)	C3—N2—C1—N1	−177.0 (3)
Hg1—S1—C1—N2	−121.0 (3)		

Symmetry codes: (i) −*x*+1, *y*, −*z*+1/2.

### Hydrogen-bond geometry (Å, °)

**Table:**

*D*—H···*A*	*D*—H	H···*A*	*D*···*A*	*D*—H···*A*
N1—H1N···S1^i^	0.80 (6)	2.67 (5)	3.415 (4)	157 (4)
N2—H2N···N3^ii^	0.79 (5)	2.21 (6)	2.951 (7)	155 (4)

Symmetry codes: (i) −*x*+1, *y*, −*z*+1/2; (ii) −*x*+1/2, *y*+1/2, −*z*+1/2.
